# The Global Status and Trends of Enteropeptidase: A Bibliometric Study

**DOI:** 10.3389/fmed.2022.779722

**Published:** 2022-02-10

**Authors:** Xiaoli Yang, Hua Yin, Lisi Peng, Deyu Zhang, Keliang Li, Fang Cui, Chuanchao Xia, Haojie Huang, Zhaoshen Li

**Affiliations:** ^1^Department of Clinical Medicine, Ningxia Medical University, Yinchuan, China; ^2^Shanghai Pudong New Area Gongli Hospital, Shanghai, China; ^3^Department of Gastroenterology, Changhai Hospital, Naval Medical University, Shanghai, China; ^4^Shanghai Institute of Pancreatic Diseases, Shanghai, China; ^5^Department of Gastroenterology, The First Affiliated Hospital of Zhengzhou University, Zhengzhou, China

**Keywords:** enteropeptidase, Web of Science, research frontier, bibliometrics, visualization

## Abstract

**Background:**

Enteropeptidase (EP) is a type II transmembrane serine protease and a physiological activator of trypsinogen. Extensive studies related to EP have been conducted to date. However, no bibliometric analysis has systematically investigated this theme. Our study aimed to visualize the current landscape and frontier trends of scientific achievements on EP, provide an overview of the past 120 years and insights for researchers and clinicians to facilitate future collaborative research and clinical intervention.

**Methods:**

Quantitative analysis of publications relating to EP from 1900 to 2020 was interpreted and graphed through the Science Citation Index Expanded of Web of Science Core Collection (limited to SCIE). Microsoft office 2019, GraphPad Prism 8, VOSviewer, and R-bibliometrix were used to conduct the bibliometric analysis.

**Results:**

From 1900 to 2020, a total of 1,034 publications were retrieved. The USA had the largest number of publications, making the greatest contribution to the topic (*n* = 260, 25.15%). Active collaborations between countries/regions were also enrolled. Grant and Hermontaylor were perhaps the most impactful researchers in the landscape of EP. *Protein Expression and Purification* and the *Journal of Biological Chemistry* were the most prevalent (79/1,034, 7.64%) and cited journals (*n* = 2,626), respectively. Using the top 15 citations and co-citations achievements clarified the theoretical basis of the EP research field. Important topics mainly include the structure of EP, the affective factors for activating substrates by EP, EP-related disorders, and inhibitors of EP.

**Conclusion:**

Based on the bibliometric analysis, we have gained a comprehensive analysis of the global status and research frontiers of studies investigating EP, which provides some guidance and reference for researchers and clinicians engaged in EP research.

## Introduction

Enteropeptidase (EP), also named enterokinase, a type II transmembrane serine protease, is localized to the brush border of the duodenal and jejunal mucosa. It is synthesized as a zymogen (proenteropeptidase) that requires activation by another protease, either trypsin or possibly duodenase. Active EP then converts the pancreatic precursor trypsinogen to trypsin by cleavage of the specific trypsinogen activation peptide Asp-Asp-Asp-Asp-Lys (DDDDK), which is highly conserved in vertebrates (1). Trypsin, in turn, activates other digestive zymogens, such as chymotrypsinogen, proelastase, procarboxypeptidase, and prolipase, in the lumen of the intestinal tract (2). The important biological function of EP is highlighted by the manifestation of severe diarrhea, failure to thrive, hypoproteinemia, and edema as a result of congenital deficiency of EP activity in the gut. Newborn infants with a congenital deficiency of the enzyme must have pancreatic enzyme replacement therapy or an amino acid mixture supplied in the diet for growth and normal health (3, 4). Conversely, duodenopancreatic reflux of proteolytically active EP may cause acute and chronic pancreatitis (5).

**Figure 1:**
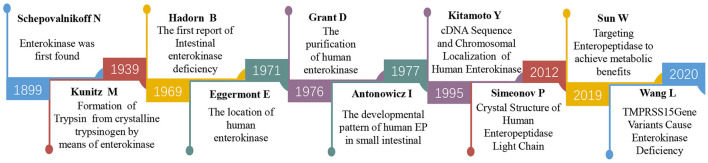
The timeline of part of the key discoveries in EP research.

EP was first discovered by Schepovalnikoff in (6). He found that duodenal secretions could activate pancreatic proteolytic enzymes, and one of these secretions was considered “an enzyme of enzymes” and named enterokinases (Figure 1), which was designated enteropeptidase by the International Union of Biochemistry (IUB). EP have been identified to be present only in the upper part of the duodenum by using histochemical staining and immunofluorescence localization techniques (7–9). In the 1930s, Kunitz partially purified the enzyme from intestinal juice and identified it as a proteolytic enzyme and the physiological activator of trypsinogen (10). During the subsequent decades, researchers have also confirmed the peptidase activation of purified EP on trypsinogen (11). To obtain more profound information about this protein, researchers attempted to acquire highly purified EP from porcine, bovine, and human populations in the following years (12–14). The purified human EP from accumulated duodenal fluid by affinity chromatography indicated that EP was an acidic glycoprotein containing 57% sugar (neutral sugars 47%, amino sugars 10%) with a molecular weight of 296,000 (13). However, there was still some contamination, principally by the α-glucosidases in the purified protein. In 1978, Grant et al. optimized the preparative procedure of EP purification by inclusion of negative affinity chromatography with glycylglycyl-anilineIn and obtained a better purity than before (15).

Protein purification further allowed the researchers to explore the structure of EP. In 1994, Kitamoto et al. first identified the chromosomal localization and cDNA sequence of human EP (16). The EP gene, located on human chromosome 21q21 and composed of a 3,696 nt cDNA sequence, contains an open reading frame of 3,057 nt that encodes a 784 amino acid heavy chain followed by a 235 amino acid light chain; the two chains are linked by disulfide bonds. The heavy chain contains seven domains. These domains resemble motifs of the SEA domain (found in sea urchin sperm protein, enteropeptidase and agrin, one copy), the LDL receptor (LDLR, two copies), complement component Clr (Clr/s, two copies), metalloprotease meprin (MRM, one copy), and macrophage scavenger receptor (MSCR, one copy) (2, 17). The enterokinase light chain is homologous to trypsin-like serine proteinases with active site histidine (H), aspartate (D), and serine (S) residues (the catalytic triad). These structural features are conserved among human, bovine, and porcine EP. The amino acid sequence determines its spatial structure. In 2012, Simeonov et al. successfully presented the crystal structures of a supercharged variant of the human EP light chain, which was the milestone that revealed the spatial architecture of the human EP (18).

The understanding of its structure helps to further explore its function. Since 1969, Hadorn et al. (19) reported the first case of intestinal EP deficiency in an infant presenting with diarrhea, failure to thrive, and hypoproteinemia edema, which provided evidence of the primary effect of EP in protein digestion. Scientists have never stopped exploring the characterization of EP and its relationships with human diseases, including acute/chronic pancreatitis, congenital enteropeptidase deficiency (EKD), tumors, and metabolic syndrome (4, 20–23). Additionally, inhibitors of EP have received tremendous attention in recent years and are expected to achieve treatment benefits from targeting EP (24).

Bibliometric research provided a practical approach to analyze the characteristics of existing literature on a subject, to identify future research directions and to improve decision-making by reducing the margin of error (25, 26). Despite the immense amount of recent research as well as the large number of review articles on EP, no bibliometric analysis on the subject has been published to the best of our knowledge. Therefore, we conducted a bibliometric study on EP publications published during the past 120 years to gain a comprehensive understanding of EP research trends from multiple aspects, in terms of annual output, productive countries/regions, scientific journals, most cited publications and references, and co-occurrence of keywords. This would enable us to provide a reference for clinical researchers and practitioners. The purpose of this study is to provide a valuable reference and guidance for researchers studying EP and to provide a novel strategy for prospective investigations.

## Methods

### Data Source

In the present study, data were retrieved by searching the Clarivate Analytics Web of Science Core Collection (WoSCC), limited to Science Citation Index-Expanded (SCIE) on August 10, 2020, to identify more than a century (1900-2020) of EP-related publications with no language restriction. Our search strategy was as follows: “Enteropeptidase” (All Fields) or “Enterokinase” (All Fields) or “Serine Protease 7” (All Fields) or “Transmembrane protease 15” (All Fields) or “TMPRSS15” (All Fields) or “ENTK” (All Fields) or “PRSS7” (All Fields). All retrieved records were downloaded in txt format on August 10, 2020, and imported into bibliometrics and visualization tools for further analysis.

### Statistical Analysis

Data on the annual output (number of publications per year), productive countries/regions (if a publication was completed by multiple countries/regions, the publication was equally distributed to all participating countries/regions), journals, top authors, and keywords were analyzed by Microsoft Office Excel 2019 (Redmond, Washington, United States). GraphPad Prism software (version 8.3.0) was used to construct the non-linear regression model. VOSviewer (1.6.17) was used to visualize the collaborations of countries/regions or authors, co-cited journals (if the journal was renamed, they will be considered as different journals in the calculation), and key references. On the VOSviewer maps, different bubbles represent elements (authors, journals, references, and keywords), and the size of the bubbles represents the number or frequency of targeting elements. The line between two bubbles reflects the relationship, and the thickness of the lines reflects the strength of the relationship between the elements (27, 28). In this research, the VOSviewer parameters were set as follows: the counting method was full counting, and the threshold (T) of the elements was dependent on the corresponding element. Utilize the R-bibliometrix and R-plot to create the geographical distribution maps of countries/regions and annual output of publications.

## Results

### Annual Output of Publications

**Figure 2:**
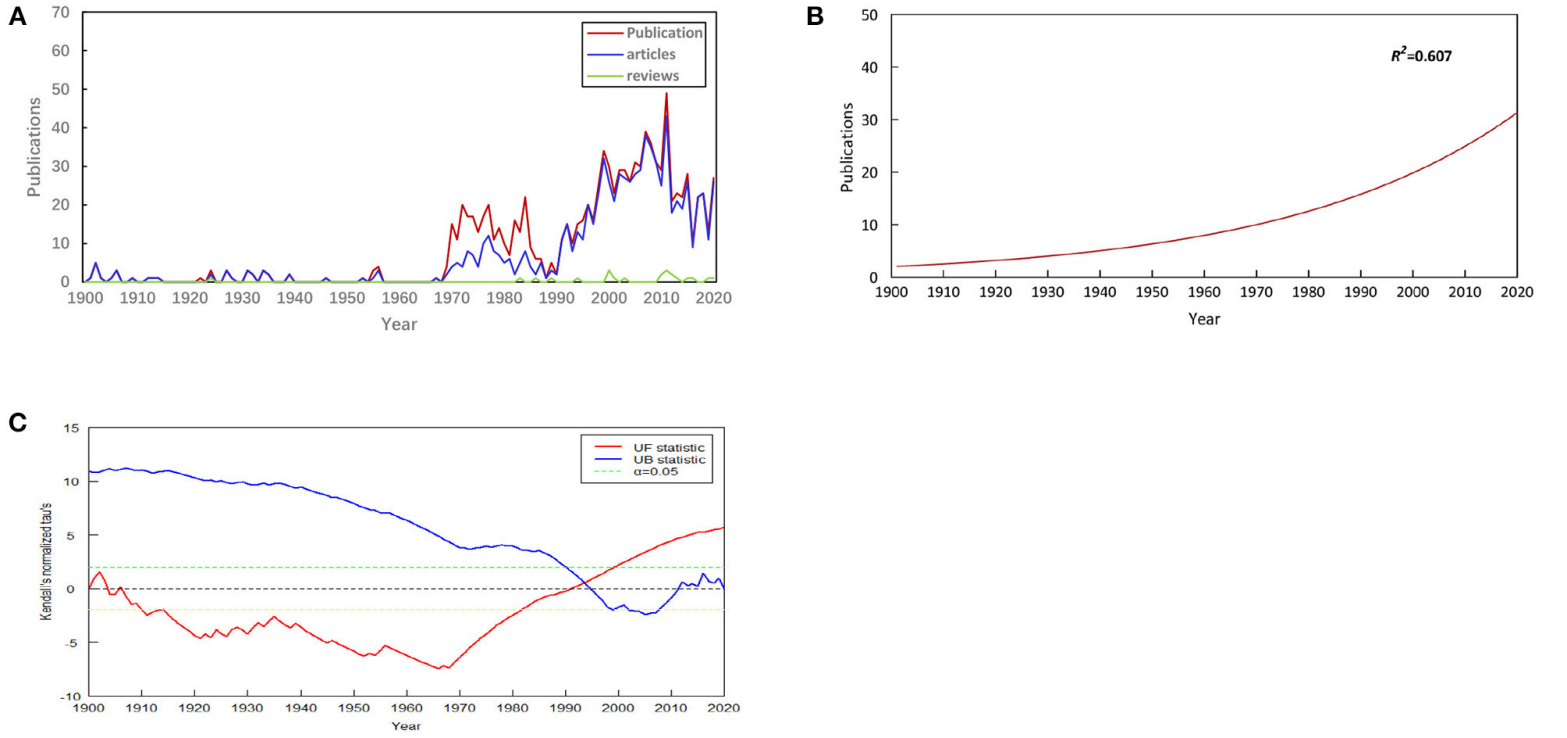
Annual output of publications. **(A)** Number of publications by year (1900-2020), **(B)** Model fitting curves of growth trends in publications, **(C)** Mann-Kendall monotonic trend test for publications.

From 1900 to 2020, a total of 1,034 publications were associated with EP in WoSCC. Among these literature types, 834 (80.66%) were categorized as “articles,” and 22 (2.13%) were categorized as “reviews.” English was the predominant language of publications in this field, denoting 94.68% (979/1,034) of the total. The most common non-English language was French, which constituted 2.13% (22/1,034) of the total, followed by Russian (14, 1.35%), German (12, 1.16%), Chinese (5, 0.48%), Czech (1, 0.10%), and Spanish (1, 0.10%). The annual publication output in the EP field is shown in Figure 2A. We can see that the overall annual output has shown an upward trend. The non-linear regression model constructed by GraphPad Prism software (version 8.3.0) also shows the steady growth of global publication output during this period (Figure 2B). To further research the status of annual output, we conducted a Mann-Kendall monotonic trend test by R-bibliometrix. The results show that there existed an obvious turning point in the change of the number of publications. The turning point occurred in ~1993 (the intersection of the UF and UB curves). The trend of the UF curve shows that the number of publications has been on the rise since 1991 and has increased significantly after 1999 (above the significance level α = 0.05) (Figure 2C).

### Country/Region Analysis

**Figure 3:**
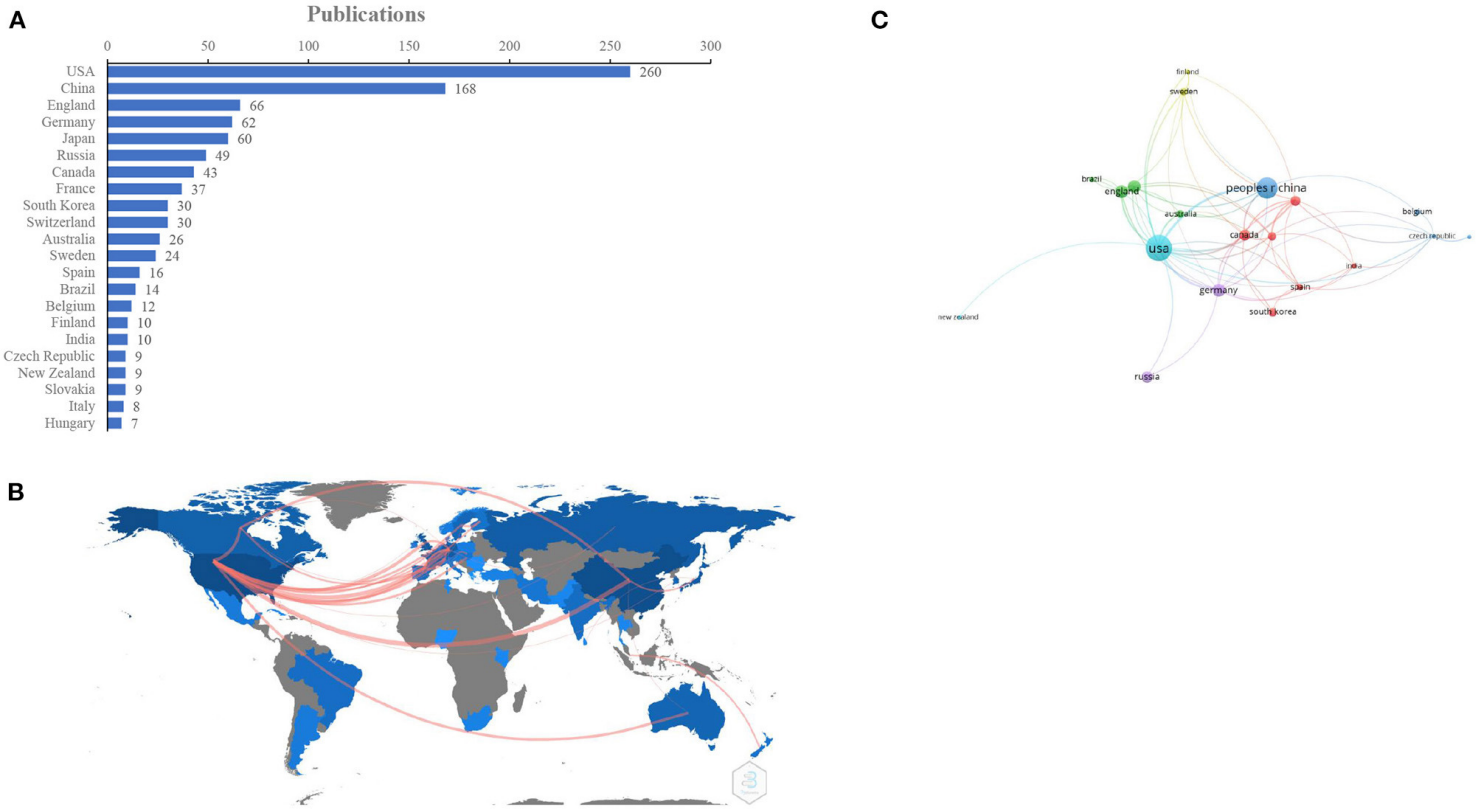
Country/region analysis. **(A)** The top 20 most productive countries/regions for EP research, **(B)** Collaborations of the countries/regions in EP field, **(C)** Network map of countries/regions (T = 9) related to EP research.

The contributions provided by different countries/regions were estimated by the number of publications. On the basis of a defined search, 56 countries/regions contributed to EP research. The top 20 productive countries/regions are shown in Figure 3A. The USA published the highest number of papers (*n* = 260), followed by China (*n* = 168), England (*n* = 66), Germany (*n* = 62), Japan (*n* = 60), Russia (*n* = 49), Canada (*n* = 43), and France (*n* = 37). The top 20 countries/regions were primarily distributed across Europe, Asia, North America and Australia; North America, and Europe were the top two highest-output regions (Figure 3B). Countries/regions (20/56, 35.71%) with ≥9 publications (T = 9) were used to construct a country/region co-authorship network (Figure 3C). The network map reflects the state of research activities and communication among these countries/regions. In Figure 3C, the USA, China, England, and Germany had larger bubbles, representing higher numbers of papers. The visualization map showed active collaborations between countries/regions; for instance, the USA had close cooperation with China, England, Germany, France, and Australia.

### Author Contributions

**Figure 4:**
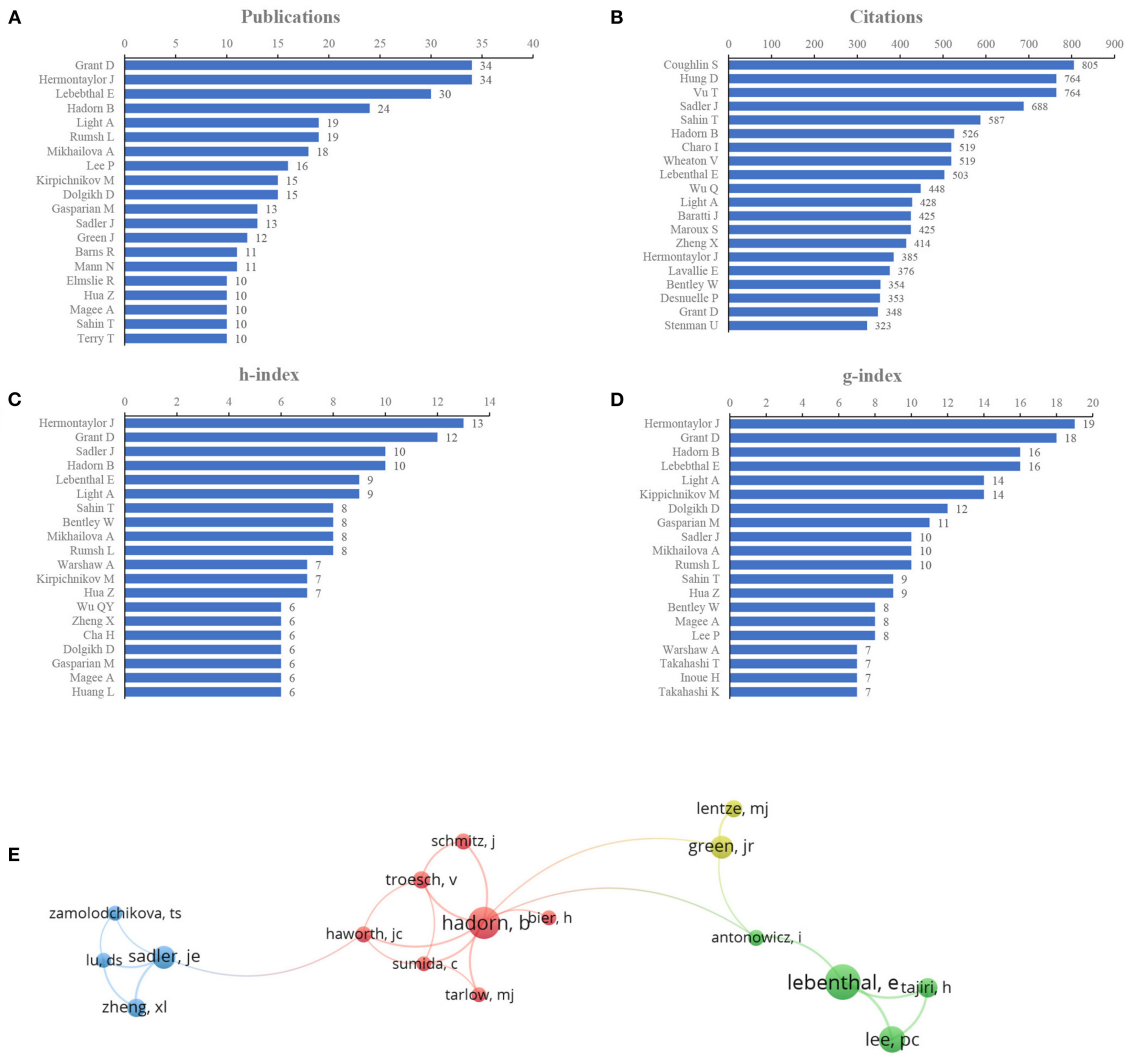
Author contributions. **(A)** Number of publications from different authors, **(B)** Total citations in the research filed from different authors, **(C)** h-index of publications from different authors, **(D)** g-index of publications from different authors, **(E)** Network map of co-authorship between authors with more than five publications.

The impactful authors were appraised by the number of publications. Grant and Hermontaylor were the most productive authors, with 34 articles (3.29% of all articles), followed by Lebenthal (30, 2.90%), Hadorn (24, 2.32%), and Light (19, 1.84%) (Figure 4A). We also evaluated the author contributions by three parameters: the citation count, h-index, and g-index. Citation count is a measure for evaluating the influence of scientists and their papers; the h-index, which is a well-known metric to determine the quality of a scientist, is calculated using the citation count of papers; and the g-index is introduced as an improvement of the h-index (29, 30). According to citations in this field, Coughlin ranked first (805 citations), followed by Hung and Vu (764 citations), Sadler (688 citations), Sahin (587 citations), and Hadorn (526 citations) (Figure 4B). Publications from Hermontaylor had the highest h-index (13), followed by those from Grant (12), Sadler and Hadorn (10, each) (Figure 4C). The g-index of publications from Hermontaylor (19) also ranked first, followed by that from Grant (18), Lebenthal (16), Hadorn (16), and Kippichnikov (14) (Figure 4D). The network map also showed collaborations from the authors (Figure 4E).

### Journal Distributions

**Figure 5:**
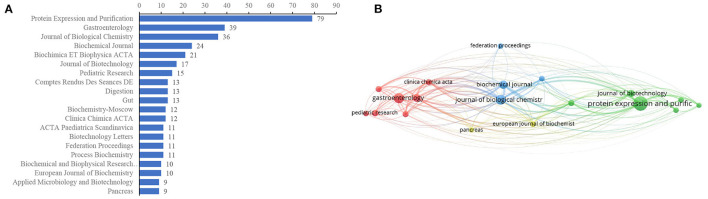
Journal distributions. **(A)** Top 20 of most prevalent journals covered by EP publications, **(B)** The network map of scholarly journals (T = 9).

The journals covered in this field were evaluated by publications and citations. Figure 5A presents the top 20 journals for EP publications. Five journals published more than 20 papers in the landscapes, of which *Protein Expression and Purification* was the most productive journal (79, 7.64%), followed by *Gastroenterology* (*n* = 39, 3.77%), *Journal of Biological Chemistry* (*n* = 36, 3.48%), *Biochemical Journal* (*n* = 24, 2.32%), and *Biochimica ET Biophysica ACTA* (*n* = 21, 2.03%). The top five journals that contributed the most to EP research accounted for 19.25% (199/1,034) of the total number of publications included in this research. Journals (19/379, 5.01%) with ≥9 publications (T = 9) were used to construct the citation network map (Figure 5B). *Protein Expression and Purification, Gastroenterology, Biochemical Journal, Journal of Biological Chemistry, Biochemical ET Biophysical ACTA*, and *Clinical Chimica ACTA* had larger bubbles representing higher journal citations. The *Journal of Biological Chemistry* had the most citations (*n* = 2,626) and had active citation relationships with *Protein Expression and Purification, Biochemical Journal*, and *Biochemical ET Biophysica ACTA*.

### Citation and Co-citation of Literatures

**Figure 6:**
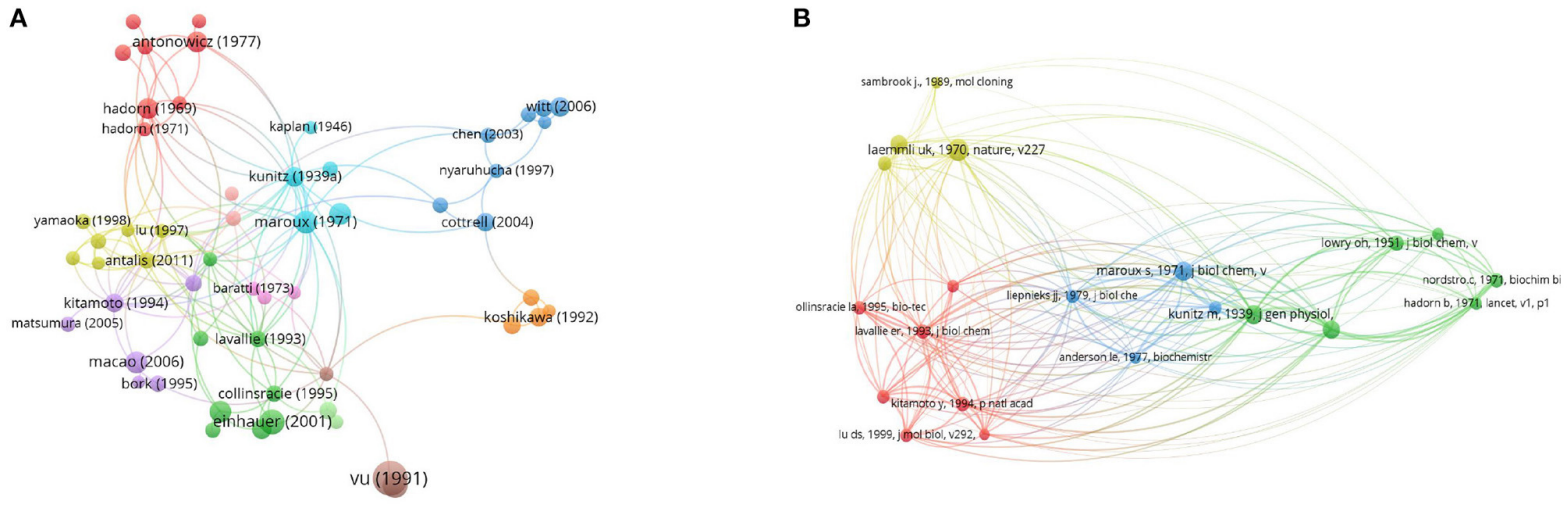
Network map of citation and co-citation literatures. **(A)** Citation analysis of publications with more than 60 citations. **(B)** Co-citation analysis of references with more than 50 citations.

The citation network map showed that 80 publications had more than 60 citations (Figure 6A). Table 1 represents the top 15 papers with the highest citations. There were 519 citations for “Domains specifying thrombin-receptor interaction” from the Journal of Nature (31), followed by “Osteopontin, a novel substrate for matrix metalloproteinase-3 (stromelysin-1) and matrix metalloproteinase-7 (matrilysin)” from the Journal of Biological Chemistry (32), with 294 citations. The third most cited article was “The FLAG peptide, a versatile fusion tag for the purification of recombinant proteins” (33), with 258 citations.

**Table 1:** Top 15 citation analysis of publications on EP research.

**Rank**	**References**	**Title**	**Source**	**Affiliation**	**Country**	**citations**
1	Vu et al. (31)	Domains specifying thrombin-receptor interaction	Nature	University of California, San Francisco	USA	519
2	Agnihotri et al. (32)	Osteopontin, a novel substrate for matrix metalloproteinase-3 (stromelysin-1) and matrix metalloproteinase-7 (matrilysin)	J Biol Chem	Maine Medical Center Research Institute	USA	294
3	Einhauer and Jungbauer (33)	The FLAG peptide, a versatile fusion tag for the purification of recombinant proteins	J Biochem Bioph Meth	University of Agriculture and Forestry	Austria	258
4	Scarborough et al. (34)	Tethered ligand agonist peptides. Structural requirements for thrombin receptor activation reveal mechanism of proteolytic unmasking of agonist function	J Biol Chem	COR Therapeutics, South San Francisco	USA	245
5	Maroux et al. (35)	Purification and specificity of porcine enterokinase	J Biol Chem	National de la Recherche scientifique	France	204
6	Kumaraswamy (36)	luorescent-conjugated polymer superquenching facilitates highly sensitive detection of proteases	Proc Natl Acad Sci USA	QTL Biosystems	USA	197
7	Waugh (37)	An overview of enzymatic reagents for the removal of affinity tags	Protein Expr Purif	National Cancer Institute at Frederick	USA	189
8	Macao et al. (38)	Autoproteolysis coupled to protein folding in the SEA domain of the membrane-bound MUC1 mucin	Nat Struct Mol Biol	Göteborg University	Sweden	186
9	Antonowicz and Lebenthal (39)	Developmental pattern of small intestinal enterokinase and disaccharidase activities in the human fetus	Gastroenterology	The Children's Hospital of Buffalo	USA	165
10	Martinez et al. (40)	Expression of recombinant human phenylalanine hydroxylase as fusion protein in Escherichia coli circumvents proteolytic degradation by host cell proteases. Isolation and characterization of the wild-type enzyme.	Biochem J.	University of Bergen	Norway	164
11	Hadorn et al. (19)	Intestinal enterokinase deficiency	Lancet	University of Berge	Switzerland	158
12	Kimura et al. (41)	The DYRK1A gene, encoded in chromosome 21 Down syndrome critical region, bridges between beta-amyloid production and tau phosphorylation in Alzheimer's disease	Hum Mol Genet	Osaka University Graduate School of Medicine	Japan	155
13	Witt et al. (42)	A degradation-sensitive anionic trypsinogen (PRSS2) variant protects against chronic pancreatitis	Nat Genet	Charité University Hospital	Germany	152
14	Yamamura et al. (43)	Molecular cloning of a novel brain-specific serine protease with a kringle-like structure and three scavenger receptor cysteine-rich motifs	Biochem Biophys Res Com	Kyoto Prefectural University of Medicine	Japan	148
15	Kunitz (10)	Formation of trypsin from crystalline trysinogen by means of enterokinase	J Gen Physiol	The Rockefeller Institute for Medical Research	USA	148

We also employed 21 references that were co-cited in more than 34 citations (Figure 6B). Among them, “Laemmli (44)” had the largest publication scale and had active co-cited corporations with “Bradford (45)” and “LaVallie et al. (46).” The top 15 references with the highest citations are listed in Table 2. The top five references with the largest number of citations were from Laemmli [(44); 118 citations], Kunitz [(10); 88 citations], Maroux et al. [(35); 88 citations], Hadorn et al. [(19); 85 citations] and Bradford [(45); 79 citations].

**Table 2:** Top 15 co-citation analysis of cited reference on EP research.

**Rank**	**References**	**Title**	**Source**	**Affiliation**	**Country**	**Co-citations**
1	Laemmli (44)	Cleavage of structural proteins during the assembly of the head of bacteriophage T4	Nature	MRC Laboratory of Molecular Biology	UK	118
2	Kunitz (10)	Formation of trypsin from crystalline trypsinogen by means of enterokinase	J Gen Physiol	The Rockefeller Institute for Medical Research	USA	88
3	Maroux et al. (35)	Purification and specificity of porcine enterokinase	J Biol Chem	National de la Recherche scientifique	France	88
4	Hadorn et al. (19)	Intestinal enterokinase deficiency	Lancet	University of Berge	Switzerland	85
5	Bradford (45)	A rapid and sensitive method for the quantitation of microgram quantities of protein utilizing the principle of protein-dye binding	Anal Biochem	University of Georgia	Georgia	79
6	Lowry et al. (48)	Protein measurement with the Folin phenol reagent	J Biol Chem	Washington University School of Medicine	USA	58
7	Kitamoto et al. (16)	Enterokinase, the initiator of intestinal digestion, is a mosaic protease composed of a distinctive assortment of domains	Proc Natl Acad Sci USA	Washington University School of Medicine	USA	54
8	LaVallie et al. (49)	Cloning and functional expression of a cDNA encoding the catalytic subunit of bovine enterokinase	J Biol Chem	Genetics Institute	UK	53
9	LaVallie et al. (46)	A thioredoxin gene fusion expression system that circumvents inclusion body formation in the E. coli cytoplasm		Genetics Institute	UK	49
10	Nordstro (47)	Rat enterokinase-effect of ions and localizationin intestine	Biochimica ET Bioph ACTA	University of Lund	Sweden	49
11	Liepnieks and Light (50)	The preparation and properties of bovine enterokinase	J Biol Chem	Purdue University	Indiana	48
12	Lu et al. (51)	Bovine proenteropeptidase is activated by trypsin, and the specificity of enteropeptidase depends on the heavy chain	J Biol Chem	Washington University	USA	48
13	Louvard et al. (52)	On the preparation and some properties of closed membrane vesicles from hog duodenal and jejunal brush border	Biochim Biophys Acta	National de la Recherche scientifique	France	45
14	Collins-racie et al. (53)	Production of recombinant bovine enterokinase catalytic subunit in Escherichia coli using the novel secretory fusion partner DsbA	Biotechnology	Genetics Institute	USA	45
15	Lu et al. (54)	Crystal structure of enteropeptidase light chain complexed with an analog of the trypsinogen activation peptide	J Biol Chem	Washington University	USA	45

### Co-occurrence of Keywords

**Figure 7:**
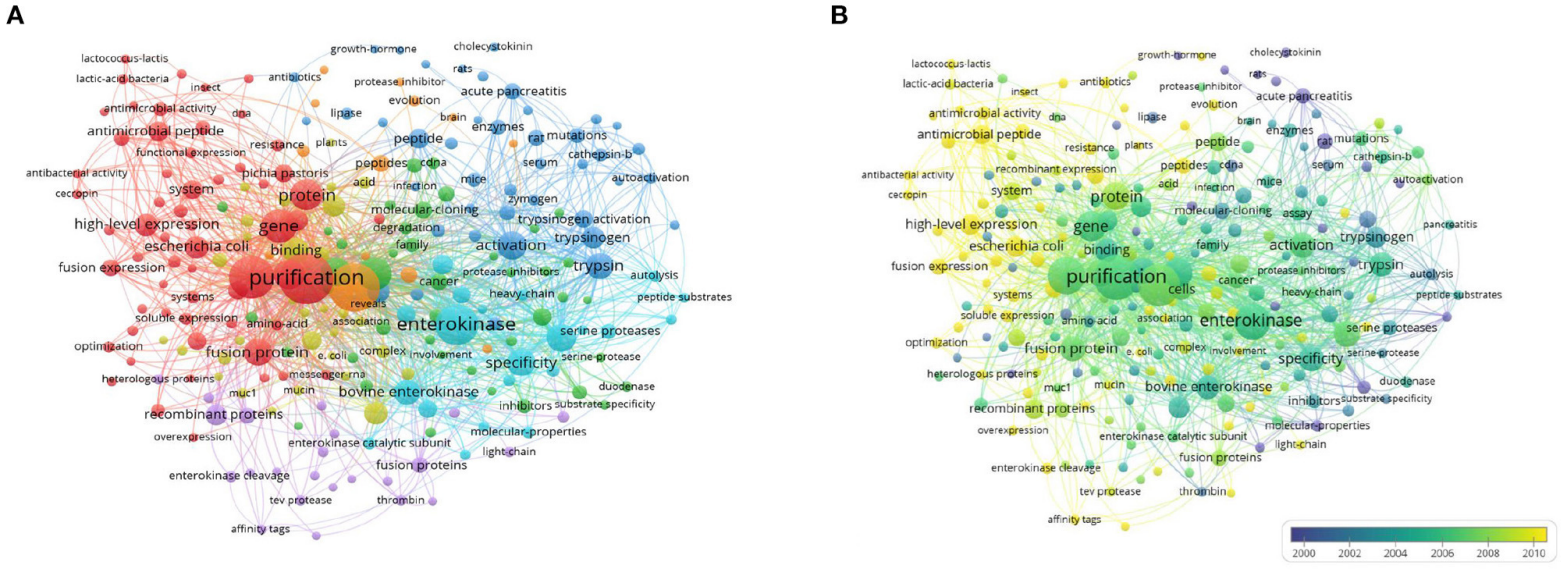
Co-occurrence analysis of keywords. **(A)** Network visualization map of keywords, **(B)** Overlay visualization map of keywords.

When two keywords appeared in the same publication, a co-occurrence relationship was formed between them. All keywords with strong co-occurring relationships can reveal research hotspots more accurately than a single keyword. We analyzed a total of 234 keywords that were identified as occurring more than five times with the full counting method and generated the network visualization map (Figure 7A) and overlay visualization map (Figure 7B). In the visualization map of keywords, the line was a symbol connecting two keywords. The size of the bubble indicated the number of occurrences. The colors displayed in the network visualization map indicate the different clusters produced by the keywords, and those in the overlay visualization map indicate the average publication year of the identified keywords.

## Discussion

### Essential Information

#### Annual Outputs, Countries/Regions, Authors, and Journals

Bibliometric analysis of the output of global publications enabled us to understand the development of EP. Based on WoSCC SCIE EP-related studies from 1900 to 2020, we performed a bibliometric analysis to fully understand the trend of EP research in the past 120 years and provide references for researchers in this field. Our study found that EP research has been conducted for more than a century, and it has experienced relatively rising progress. In nearly 60 years, the annual output before 1969 did not exceed five publications. Considering the relatively slow transmission of economic development in those years, this situation was understandable. The current analysis showed that the number of publications on EP has increased rapidly since 1999, which may be related to rapid economic growth and the fact that many researchers in different disciplines are active in the field. Overall, the annual output related to EP research followed an upward trend during the investigated period, which indicated that EP research has received increasing attention in recent years. The USA, China, England, Germany and Japan were the top five productive countries, indicating that these countries are particularly influential in the field of EP research. Among the top 20 countries, North America and Europe have contributed the most in publications, demonstrating that these two regions may be potential areas for EP research and development. The country collaboration map and network map illustrated the relationships of these top contributed countries/regions, which fully explored EP research as a worldwide activity. This kind of cooperation between countries/regions worldwide may bring academic sharing, attract better scientists engaged in the field and accelerate EP-related research.

Analyses of authors help to understand a research field more comprehensively and objectively evaluate the contributions of researchers, as well as their research level and academic status in this field (55). The top 20 authors with the most publications were active researchers in this theme. Grant produced the largest number of papers, Coughlin has the most citations per paper, and Hermontaylor currently has the highest h-index and g-index. These data and indicators provide us with the most academically influential and authoritative authors in the EP field. Significantly, most of the authors were from institutions in universities and hospitals. Some researchers majored in chemistry, biology, or biochemistry, and some majored in biophysics and medicine. Grant and his colleague Hermontaylor, both from the Georges Hospital of St Georges University, made great contributions to EP research, including the functions, location, and purification in humans and porcine (5, 13). Coughlin was more concerned about the applications of EP (34). We also analyzed the co-authorship of more than five publications and found that there was cooperation and communication among authors, but not closely. Therefore, future research should focus on author cooperation. In other words, identifying these leaders would help us to review and analyze the literature before starting new EP-related research and help us understand the basic information of this field precisely and quickly.

The distributions of journals are a basic part of a bibliometric analysis. We analyzed the main scientific journals that have published on this subject. The results showed that *Protein Expression and Purification, Gastroenterology, Journal of Biological, Chemistry Biochemical Journal*, and *Biochimica ET Biophysica ACTA* were the most prevalent journals, and the co-cited frequency of network map presented journals were intimately connected with each other in the field. which may provide a reference for beginners to conduct research and submit a novel discovery in this field. These journals mainly cover basic fields, indicating that EP studies were primarily basic studies. We should pay more attention to the relationship between EP and clinical diseases in the future, which may help to better carry out clinical transformation from basic theoretical knowledge and provide better services for life sciences.

### Research Status of EP

#### Citation of Publications

The number of citations of a study may represent its most important bibliometric quality because it represents the relevance and importance of a study in the academic world. In the present study, we reviewed publications with citations from 1900 to 2020. Among the 1,034 publications, 80 (7.74%) documents were cited more than 60 times and were included in the citation analysis. The 15 top-cited publications listed were quite different. Overall, five articles were concerned about the structure of EP; four of fifteen were concerned about the expression, recombination, and properties of EP; three of these studies were focused on the application of EP; and three documents were related to clinical diseases.

Since the discovery of EP, scientists have tried a great diversity of methods to purify EP from situ or express recombinants, which are expected to explain the functions of EP in depth. The enzyme activity properties of EP and its role in hydrolyzing trypsinogen had been indicated by researchers (10, 35, 42), which drive EP-related research to a large extent. Owing to its high specificity and effectiveness in protein hydrolysis, EP have been utilized in protein construction, cleaving the specific sites of fusion proteins. Affinity tags are often exploited to facilitate the expression and purification of recombinant proteins, every affinity tag, whether large or small, has the potential to interfere with the structure and function of its fusion partner. Therefore, reliable removal of affinity tags is needed sometimes. By fusing the target sequences of DDDDK to the C-terminal end of the selective fusion label to obtain a highly selective fusion label for immunoaffinity chromatography, and finally utilizing the EP to remove the label from therapeutic proteins, which expedited the studies of antisera against a desired protein (33, 40). Waugh reviewed the enzymes commonly used to remove affinity tags and discussed the benefits and disadvantages of EP as specific affinity tags, which greatly contributed to researchers selecting optimal enzymes according to their target proteins and the experimental objective (37). High purity protein was conducive to the study of specific domains, and fully understand the domain characteristics of protein is essential to reveal its mechanism. Investigators have used the LDPR/S domain of EP to replace this cleavage site of the platelet thrombin receptor and created a functional EP receptor, demonstrating that all information necessary for receptor activation is provided by receptor proteolysis (31). Further to explore the amino acid function in specific sites of the LDPR/S domain have contributed to reveal the details of the thrombin receptor proteolytic triggering mechanism. Thrombin and EP are both members of the serine protease family and have similarities in the LDPR/S domain, which gave us an indication that EP may have similar mechanism in this domain (34). Not only the LDPR/S domain but also SRCR and SEA domains had been investigated in different serine protease. Brain-specific serine protease (BSSP-3), a new member of the SRCR superfamily, its top four protease domains in descending order had 38.0% similarities with human EP (43). Most of the SRCR superfamily members are known to be expressed on the surface of T cells, B cells and macrophages involved in the immune system and host defense functions (43). Macao B found that SEA domains may have a role in autoproteolysis by conformational stress and conserved serine hydroxyl groups (38). From the above reported highly cited publications, the relative research of EP was almost focused on the cleavage site, single domain properties and enzyme activation of EP in the basic field, which provided a rich research basis for our follow-up research on EP.

In 1969, Hadorn et al. first reported the case of intestinal EP deficiency in an infant and illustrated that EP was closely related to clinical disease (19). Later, in 1977, Antonowicz et al. clarified the developmental pattern of small intestinal EP and disaccharidase activities in the human fetus (39). This provided the theoretical basis for the clinical intervention of patients with congenital EP deficiency. With the increasing amount of evidence, researchers found that EP was associated not only with gastrointestinal illnesses but also with other diseases, such as Alzheimer's disease, which was found by Kimura et al. and may be associated with the EP gene in 2007 (41). However, the exact mechanism of EP with diseases needs further study.

#### Co-citation of References

The co-cited references represent the frequency of two publications being cited together by other publications (27). In our study, the top co-cited references were used to investigate the knowledge base for the EP landscape. The top 15 co-cited references were selected to identify the knowledge base related to EP. Overall, almost all of these references were basic research, and only one study was clinical research (19); one-third of the top co-cited references were focused on techniques including protein identification, recombination and expression (44–46, 48, 52); six-fifteenths of the references were more focused on the structures and characteristics of EP (16, 49–51, 53, 54).

“Intestinal enterokinase deficiency” by Hadorn et al. (19), which was one clinical study, and the first case that was reported about an infant presenting with diarrhea, failure to thrive, and hypoproteinemia edema was shown to have deficiency of intestinal EP, which resulted in the failure to activate pancreatic proteolytic enzymes, emphasizing the significance of EP in digestion and activation of proteolytic enzymes in the pancreas. Other studies were more focused on basic research. For instance, the studies of Laemmli, Bradford, LaVallie et al. and Liepnieks and Light in the top co-citations list reported the techniques of protein expression and separation techniques, which provides great technical support for the bioengineering development of EP (44–46, 49, 50). Kunitz and Maroux et al. utilized the purified protein to verify the catalytic properties of EP to trypsinogen (10, 35). Notably, LaVallie et al. developed an expression method for cloning and expressing cDNA encoding the catalytic subunit of bovine EP and indicated that in the absence of the non-catalytic EP heavy chain, the recombinant light chain of EP was also capable of activating trypsinogen, which fully explained the catalytic function of the EP light chain (49). Previous studies have paid more attention to catalytic chains instead of heavy chains and considered heavy chains to play a role in anchoring. In 1997, Lu et al. illustrated that bovine EP containing heavy chain and light chain cleaved trypsinogen at pH 5.6 with 520-fold greater catalytic efficiency than did single light chain; additionally, they found that the heavy chain has little influence on the recognition of small peptides but strongly influences macromolecular substrate recognition and inhibitor specificity (51). He first described the function of EP heavy light experimentally and discussed the interaction between the heavy chain and light chain. Two years later, he parsed the crystal structure of bovine EP light chain complexed with an analog of the trypsinogen activation peptide, which veiled a new journey of structural research in the EP field.

### Perspectives

This research presented some remarkable viewpoints about EP indexed by the WoSCC database between 1900 and 2020. The keyword co-occurrence analysis showed the results obtained by publications under a wide range of bibliometric indicators, which could enable the identification of hotspots and trends and guide researchers to emulate related topics in the field. In Figure 7, as seen from the network map, the co-occurrence of keywords was mainly clustered into four research fields with EP. In this study, we summarized the hotspots of EP research as follows.

#### Spatial Architecture of EP: Lack of Structure of the EP Heavy Chain

The structural features of EP are well conserved among various species of vertebrates. In 1999, Lu et al. resolved the crystal structure of bovine EP light chain complexed with an analog of the trypsinogen activation peptide with 2.3 Å resolution (54). They found that the lysine (Lys) at substrate position P1 is conserved with other trypsin-like serine proteases, while the aspartyl residues at positions P2-P4 of the inhibitor interact with the enzyme surface mainly through salt bridges with the N atom of Lys99. The Lys99 mutation may specifically prevent the cleavage of trypsinogen and binding with small-molecule inhibitors. Human enzymes show 10 times faster kinetics than those of other vertebrates, but low solubility under low salt conditions that hampers protein production and crystallization (56, 57). Therefore, it was not until 2012 that Simeonov et al. identified the crystal structure of a supercharged variant of the human EP light chain (18). A supercharged variant (N6D/G21D/G22D/N142D/K210E/C112S) of human EP that does not affect the structural integrity of the protein was constructed to increase the solubility to use for crystallization. The structure (resolution, 1.9 Å) displays a typical α/β trypsin-like serine protease fold. Overall, the solving of the light chain of bovine and human EP with high resolution by crystallization has promoted the process of research on the spatial structure of EP and provided a strong foundation for subsequent research in this field. In 1997, Lu et al. found that full-length EP (using the signal peptide instead of the transmembrane domain) cleaved trypsinogen at pH 5.6 with 520-fold greater catalytic efficiency than a single light chain (51), indicating that the heavy chain participated in substrate catalysis. However, the spatial structure of the EP heavy chain has not been investigated until now. Due to the very high catalytic efficiency with heavy chain participation, the low solubility, and the single-pass transmembrane nature of EP, it is difficult for researchers to resolve the full-length special structure of EP, especially the human EP. However, the technical advancements of highly accurate protein structure prediction with AlphaFold2 and cryo-electron microscopy (cryo-EM) techniques may contribute to resolving the integral special structure of EP in future years.

#### Affective Factors for Activating Substrate by EP: Ca^2+^, pH Value, Temperature, and Others

The influence of Ca^2+^ on the efficiency of hydrolysis of different substrates catalyzed by EP has been studied over time; contradictory results have been obtained by different authors for different substrates. An earlier study revealed that low calcium concentrations (<1 mM) increase EP efficiency toward this natural substrate (58), while high concentrations (>1 mM) lead to the inhibition of activation (59). Grant at the same time showed the activating effect of calcium ions: hydrolysis of GD4K-Nfa by human EP at pH 8.4 in the presence of 10 mM Ca^2+^ was three times faster than that of 0.1 mM Ca^2+^ (60). Mikhailova et al. testified that calcium ions at concentrations exceeding 10 mM indeed have an activating effect (three-fold) on hydrolysis of the substrates (glycyl-tetra-L-aspartyl-L-lysine-X) by natural full-length EP. In contrast, the hydrolysis of substrates with one or two Asp/Glu residues at P2-P3 positions is slightly inhibited by Ca^2+^. In the case of the EP light chain as well as the enzyme containing the truncated heavy chain (466–800 fragment), the activating effect of calcium ions was not detected for any of the studied substrates. To achieve high EP activity toward its natural substrate trypsinogen and retain the high specificity of hydrolysis simultaneously, the participation of one more secondary substrate binding site (SII) located in the region between 118 and 465 of the heavy chain is required. In this way, there is a strict hierarchy of the secondary substrate binding sites [i.e., one provides specificity, whereas the other provides efficiency of hydrolysis (61)]. The answer to this question can be obtained only as a result of a planned series of experiments with mutant trypsinogen forms.

The enzymatic activity of recombinant human EP light chain was active over a broad range of pH 6.9 with optimum activity at pH 7.5, and it demonstrated high stability to different denaturing agents (56). Crystalline trypsinogen is most readily and completely transformed into trypsin by means of EP in the range of pH 5.2–6.0 at 5°C and at a concentration of trypsinogen of not more than 0.1 mg/ml; the catalytic action of EP on crystalline trypsinogen in dilute solution at pH more alkaline than 6.0 and in concentrated solution at pH even slightly below 6.0 is complicated by the partial transformation of the trypsinogen into inert protein which can no longer be changed into trypsin even by a large excess of EP (10). Furthermore, enzyme catalysis is also affected by temperature and enzyme/substrate concentration. Most of the above experiments were based on catalytic light chains or the protein/substrate of animals, lacking related researches about affective factors for activating substrate of full-length human EP. Further studies should pay more attention to the molecular properties of full-length human EP *in vivo* and *in vitro*.

#### EP With Diseases: Congenital Enteropeptidase Deficiency, Pancreatitis, and Others

EP is an essential enzyme in food digestion and the physiological activator of trypsinogen, and the trypsin produced activates other zymogens, forming a mixture of proteolytic enzymes (2). EP deficiency is a rare autosomal recessively inherited disorder in the intestine that may lead to serious diarrhea, failure to thrive, and hypoproteinemia edema and malnutrition in infants (4, 62). Hollinger et al. analyzed three EKD patients from two families. They found compound heterozygosity for nonsense mutations (S712X/R857X) in two affected siblings, and compound heterozygosity for a nonsense mutation (Q261X) and a frameshift mutation (FsQ902) in the third patient. In accordance with the biochemical findings, all four defective alleles identified are predicted null alleles leading to a gene product not containing the active site of the enzyme (63). The four variants were mutations in the EP gene that can be retrieved in the Human Genome Mutation Database (HGMD). Beyond that, Wang et al. enriched the EP gene mutation map and reported two novel compound heterozygous variants in the EP gene: the c.1921G > A (E641K) variant caused the skipping of exon 16, but it is predicted to be benign and may not manifest a severe phenotype in childhood; the c.2396T > A (p. V799D) change in the serine protease domain decreased the total expression level of EP by 29%, and the EP activity of V799D mutants was decreased by 37% compared with that of wild type (4). Although EKD is considered a rare disease, the reported patients responded well to pancreatic enzyme replacement therapy. However, owing to the deficiency of genetic testing of infants with severe diarrhea, the incidence rate of EKD may be reduced to a certain extent. Therefore, we need to strengthen the genetic testing of children with severe diarrhea and clarify the specific etiology. Additionally, all above studies were focused on the EKD in infants and lacked evidence on the effects of EP mutation in adults and related disease, and thus further studies should pay more attention to this direction.

Excessive active protease may also lead to organ and tissue damage. Evidence have shown that combined caerulein/EP infusions resulted in the transformation of mild to necrotizing pancreatitis in rat models (64). The reflux of EP into the gland could trigger the inappropriate activation of the stored zymogens, which may be associated with acute necrotizing pancreatitis (5). Additionally, endoscopic retrograde cholangiopancreatography (ERCP) will interject duodenal juice that rich in EP, into the pancreatic-biliary tract, which causes inappropriately activation of trypsinogen, thereby initiating acute pancreatitis (65). However, all the above studies are based on increasing EP in the intestinal or pancreatic-biliary tract and absence of clinical evidence that EP activates trypsinogen *in situ* in the pancreas. Therefore, further research is needed to provide more direct and powerful evidence to authenticate the correlations between EP and pancreatitis.

EP may also play a role in other diseases. Recent evidence has shown that EP may also participate in body metabolites and mediate the regulation of microbiota and enterobacterial metabolites, and the EP inhibitors have shown therapeutic effects in metabolic syndrome or obesity (22, 24). A case–control study from Japan recruited 374 Japanese patients and 375 population-based controls scanned throughout chromosome 21 to assess genetic associations with late-onset alzheimer's disease and found the marker of the EP gene linked to the SNPs of chromosome 21 in late-onset alzheimer's disease patients (41). Other studies also found that the EP gene may be implicated in Down syndrome, cardiometabolicity and cancer (66–68). Since the large scope of EP covers diseases and we know little about it, extensive research is still needed to determine its relevance to specific diseases.

#### Inhibitors of EP: Classifications, Mechanisms, and Limitations

Different species of EP respond differently to different inhibitors. Light and Janska demonstrated that bovine EP was inhibited by bovine pancreatic trypsin inhibitor (BPTI), suggesting that its active site has several features in common with that of trypsin (69). BPTI exhibited greater affinity for bovine EP, which contains heavy chains and light chains than a single light chain, while soybean trypsin inhibitor (STI) could only inhibit the catalytic light chain but not the full-length bovine EP (51). Furthermore, the human and porcine EP were not inhibited by BPTI and STI (69). To better inhibit EP activity, researchers have developed a series of serine protease inhibitors, such as gabexate mesylate, camostat mesylate, nafamostat mesylate, leupeptin, and upamostat (70–73). Although they have been widely used in the clinical intervention of pancreatic diseases and other disorders, they are not specific EP inhibitors. In 2019, Sasaki et al. reported the discovery of a novel reversible inhibitor of EP, SCO-792 (74). However, SCO-792 appeared to have some potential limitations. As we reviewed above, the heavy chain of EP may participate in efficient catalysis and inhibitor specificity, and the inhibitory effect in different species may respond differently. Due to the lack of knowledge on the full-length structure and functions of EP, the discovery of EP inhibitors and the development of EP-related clinical drugs are still a long haul ahead.

## Limitations

In this study, we quantitatively analyzed the existing EP-related literature by bibliometric methodology objectively. The findings and suggestions may help researchers and clinicians understand the performance and trends of EP globally. Nevertheless, due to the inherent limitations to the bibliometric approach, we did not examine individual article records, except for those random samples used to verify the accuracy of indexing. Additionally, we only retrieved publications in the WoSCC (SCIE) database; other databases, such as PubMed, Embase, and Cochrane Library, were not searched, which may lead to some missing publications. Furthermore, we only analyzed articles with high total citations since publication instead of the recent highly impactful ones, so that certain recent highly impactful articles have not yet acquired comparable citations with older articles with high citations, which can undermine their significance. Therefore, it is still necessary to observe the latest published achievements. Despite these limitations, this research provides a solid global perspective on EP research over the past 120 years and the research direction for better scientific contribution.

## Conclusion

This bibliometric analysis has brought together the knowledge on publications related to EP that is available from the Web of Science database. Research results and recommendations indicate that the USA ranks first in productivity, and it cooperates closely with other countries, such as China, England, and Germany. Grant and Hermontaylor may be critical researchers in the field of EP. The *Protein Expression and Purification* and *Journal of Biological Chemistry* ranked first in the productive journals and cited journals, respectively. Using the top 15 citations and co-citations achievements clarified the theoretical basis of the EP research field. The key research topics identified in this study included the structure of EP, the influencing factors for activating substrates by EP, EP-related disorders, and inhibitors of EP. The topics of EP are worthy of continued follow-up by researchers.

## Data Availability Statement

All datasets presented in this study are included in the article/supplementary material.

## Author Contributions

ZL and HH designed this study. XY, HY, and LP performed the search. DZ and KL collected data. FC and CX rechecked data. XY and HY performed analysis. XY and LP wrote the manuscript. All authors read and approved the final manuscript.

## Funding

This research was supported by the role of Piezo1 protein in the angiogenesis of gastric cancer (Grant Number XM2019094). Research on demonstration application of collaborative network construction in clinical medical research, No. 2015BAI13B08, China.

## Conflict of Interest

The authors declare that the research was conducted in the absence of any commercial or financial relationships that could be construed as a potential conflict of interest.

## Publisher's Note

All claims expressed in this article are solely those of the authors and do not necessarily represent those of their affiliated organizations, or those of the publisher, the editors and the reviewers. Any product that may be evaluated in this article, or claim that may be made by its manufacturer, is not guaranteed or endorsed by the publisher.
